# Kimura's Disease in a Caucasian Female: A Very Rare Cause of Lymphadenopathy

**DOI:** 10.1155/2014/415865

**Published:** 2014-05-14

**Authors:** Ewa Osuch-Wójcikiewicz, Antoni Bruzgielewicz, Magdalena Lachowska, Agata Wasilewska, Kazimierz Niemczyk

**Affiliations:** Department of Otolaryngology, Medical University of Warsaw, Banacha 1a Street, 02-097 Warsaw, Poland

## Abstract

*Introduction*. Kimura's disease is a rare chronic inflammatory disorder characterized by the head and neck lymphadenopathy often accompanied by eosinophilia and elevated serum IgE. It is benign condition with unknown etiology usually affecting young men of Asian race. Affected Caucasians are very rare. *Case Presentation*. We report a clinically and histopathologically typical case of this disease in a 40-year-old Caucasian female. In differential diagnosis particular attention has been paid to angiolymphoid hyperplasia with eosinophilia and neoplasms. *Conclusion*. The diagnosis of Kimura's disease can be very difficult and misleading; it is important not to ignore histopathological features. The presented patient has been under follow-up with no more symptoms of the disease for the last 1.5 years.

## 1. Introduction

Kimura's disease is a chronic inflammatory condition presenting as multiple painless solitary subcutaneous nodules localized mostly in the region of the head and neck with coexisting lymphadenopathy and peripheral eosinophilia. This quite rare condition is found almost exclusively in Asian individuals in their 2nd to 4th decade of life mostly in males (70–80%) [1, 2]. The etiology is unknown. However, an allergic reaction or an alteration of immune system is taken into consideration. Persistent antigenic stimulation following arthropod bites and parasitic or candida infection are also suspected [3]. Initially the lesion was described in 1937 as a neoplasm (Kimm and Szeto). There are very rare cases of Kimura's disease reported in Caucasian individuals in Europe, United States of America, and Australia. To the best of our knowledge, the case presented in this paper is the second one reported in Poland [4].

## 2. Case Presentation

This retrospective study presents a 55-year-old Caucasian female (Polish) with a nontender nodule on the right side of her neck. The nodule was localized in the left occipital triangle on the border of the fifth level of the lymph nodes and the trapezius muscle. In addition, lymphadenopathy of the middle jugular lymph nodes (third level) on the opposite side was detected.

The nodule size was 20 mm, firm, and not fixed to the skin or any underlying tissue. It was mobile and nontender. The overlying skin was unaffected and without any pruritus or redness.

USG exam of the neck revealed an enlarged inflamed lymph node (11 mm in size) and two more smaller ones above it with 5 and 6 mm diameter in the left occipital triangle. Moreover, in the right submandibular triangle an 8 mm lymph node was described. No enlarged lymph nodes in the other cervical regions were detected. On the left side, a nodule oval in shape with a size of 13 × 14 mm was reported in the residual thyroid and described as adenoma.

The patient medical history was inconclusive. She had undergone a left-sided strumectomy at the age of 33.

Blood test results revealed a leukocyte count of 10.2 × 10^9^/L with an eosinophil rate of 25% (normal range is 1–5%), elevated protein level of 8.3 g/dL (normal range is 6.2–8.2), potassium level of 5.04 mmol/ L (norms are 3.6–5), and elevated cholesterol levels and triglycerides.

**Figure 1:**
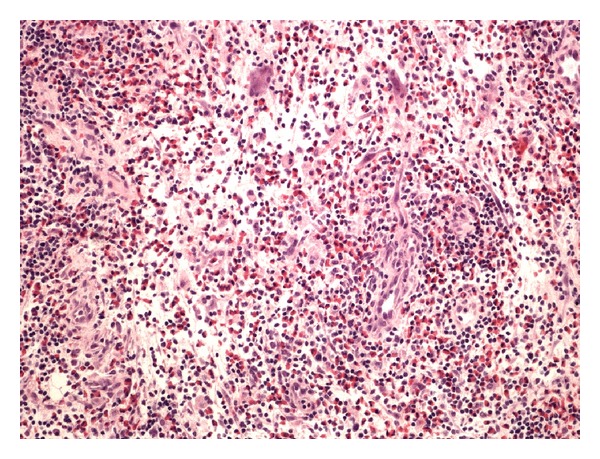
Large quantity of eosinophilic granulocytes, growth of small venules (H&E staining, microscopic magnification of ×40).

**Figure 2:**
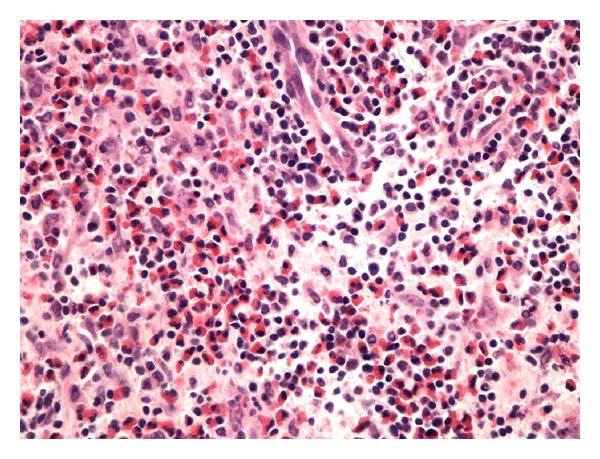
Activated lymphatic nodule, fibrosis (immunohistochemical staining with CD20 (B-cells), microscopic magnification of ×20).

Excisional biopsy was performed. Histopathology revealed a 30 mm diameter lymph node. Well-formed follicles and reactive germinal centers indicated that the node's architecture was preserved. Interfollicular zones were infiltrated with mixture of inflammatory cells, mostly eosinophils and lymphocytes and also dendritic cells, histiocytes, and immunoblasts (Figures 1 and 2). These histological features were consistent with Kimura's disease description.

The patient underwent a consultation with the oncologist. As a result, the neoplasm was excluded from differential diagnosis.

The patient has been followed up in Outpatient Department and has been asymptomatic for the last 18 months. She did not require any medications or any other medical treatment so far.

## 3. Discussion

Kimura's disease is a chronic inflammatory condition presented as painless solitary or multiple subcutaneous nodules, asymmetric, mostly in the head and neck region with coexisting lymphadenopathy in 30–40% of the cases [1]. Typical areas for the nodules are preauricular, submandibular, and popliteal regions as well as oral cavity, larynx [5], and parotid glands. They are rarely reported in other localizations like eyelids, lacrimal glands, orbit, axilla, groin, forearm, and kidneys [1, 2, 6–8].

Kimura's disease may affect kidneys in up to 60% of patients. In those cases, it may present itself as almost all types of glomerulonephritis or as nephritic syndrome (12%) [2, 5].

Hypereosinophilia and elevated serum IgE are found in Kimura's disease as well.

Kimura disease may be easily mistaken for a malignant disorder (acute lymphocytic leukemia, T-cell lymphoma, Kaposi Sarcoma, Hodgkin's disease, or parotid tumor) because of a mass localized in the parotid gland and accompanied by lymphadenopathy. That is why differential diagnosis should be performed very carefully taking into account all clinical and histological findings [2, 3, 9].

Differential diagnosis between Kimura's disease (KD) and angiolymphoid hyperplasia with eosinophilia (ALHE) has been a challenge for a long time. They were considered to be variations of the same disease making the diagnostic process very complicated.

Histologically Kimura's disease presents as preserved lymph node architecture with reactive and prominent germinal centers. Dense eosinophilic infiltration of the interfollicular zones, lysis of the follicles, and occasionally microabscesses are seen. Granuloma formations contain infiltration of eosinophils, lymphocytes, plasma cells, and histiocytes. Tissue fibrosis, sclerosis, and vascular proliferation are also present. Vessels remain thin-walled with cubical endothelial cells present. Rarely the features include progressive destruction of germinal centers, presence of polykaryocytes (which are not pathognomic for that disease). Immunofluorescence tests show germinal centers containing heavy IgE deposits and variable amounts of IgG, IgM, and fibrinogen [1–3].

As previously mentioned, Kimura's disease (nodules localized in head and neck, accompanied with lymphadenopathy) is often confused with angiolymphoid hyperplasia with eosinophilia (ALHE) that predominantly affects middle-aged Caucasian females. Histological differences are not distinct. Blood vessels walls containing hypertrophied and sometimes vacuolated endothelial cells with eosinophilic cytoplasm and rarely atypical nuclei never appear in KD. ALHE is now considered as a type of histiocytoid hemangioma. Other differences between those two diseases are presented in Table 1 [3, 10].

Ultrasound imaging of the salivary glands and neck should be the first test that is performed in case of lymphadenopathy. Lymph nodes in KD are hypoechoic, solid, and round or oval in the parotid and submandibular areas with normal surrounding soft tissues.

On radiological examination, Kimura's disease mimics other chronic and malignant diseases such as tuberculosis or lymphoma. In case of a mass in the major salivary gland, the differential diagnosis should include the following: adenocarcinoma, adenoma, and metastatic lesions. Despite the similar radiological characteristics, in case of Kimura's disease it is not possible to exclude malignancy and thus diagnosis cannot be based exclusively on imaging. A histological confirmation is necessary. Preoperative USG, CT, and MRI are useful in demonstrating salivary gland involvement and localization of abnormal lymph nodes and to assist in guided biopsy [11].

There is no consensus on the management aspects in Kimura's disease so far. However, primary prophylactic surgery is performed as a therapeutic and/or diagnostic procedure. Conservative treatment includes oral steroids which are reported to be responsible for decreasing size of the enlarged lymph nodes but there is no evidence of reduction of the affected salivary gland size. Furthermore, the lesions usually get enlarged again when steroid treatment is terminated. Thus successful treatment is mainly reassured by a constant low dose of steroids [2, 3, 10, 12, 13]. Another positive effect of steroid treatment is that it decreases renal symptoms as well.

Remissions reach 25% in groups of patients treated surgically. Surgery and following subsequent steroid treatment are proposed as an alternative treatment [3]. Radiation therapy is useful to control lesions that are not responsive to steroids or with a relapse after surgery. Effective total dose of radiation is proved to be 20–30 Gy [14].  Table 2 presents indications for different treatment options in case of Kimura's disease.

Besides traditional therapy, many other treatment methods have been studied. These methods include retinoids, immunosuppressants, monoclonal antibodies (imatinib), antiallergenic drugs (suplatast tosilate and cetirizine), leukotriene-receptor blocker (panlukast), and pentoxifylline [15] with variable effect [11].

## 4. Conclusions

Kimura's Disease is a chronic benign inflammatory condition endemic to Asians. The case presented in this paper is an exceptional case described in a Caucasian female (Polish). Other than the rarity of this disease in the Caucasian population, to the best of our knowledge, it is the second case reported in Poland so far. If not properly diagnosed, the cervical lymphadenopathy in Kimura's disease may be initially mistaken for a malignancy. However, due to a well-obtained clinical history and histopathologic awareness a proper diagnosis had been established in that case. Our patient has been asymptomatic for the last 18 months without any relapse.

## Figures and Tables

**Table 1:** Comparison of KD and ALHE.

	Kimura's disease	ALHE
Demography	Japan, Korea, China	Without limits, mostly Caucasian race
Age	2-3 decades	3–5 decades
Sex	Mostly men	Mostly women
Location of lesions	Head and neck mostly	Various, head and neck
Characteristic of lesions	Deep subcutaneous nodules (>2 cm), no skin changes	More superficial papules, nodules, and tumors (<2 cm), erythematous to brown skin
Lymphadenopathy	Common	Uncommon
Serum IgE	Usually elevated	Usually normal
Glomerulonephritis	Sporadically	Exceptionally

**Table 2:** Kimura's disease management options along with indications [2]. (1) Once diagnosed rule out renal involvement first. (2) Choose one of the following treatment options according to presented indications as follows.

Surgery	Medical (steroids, immunosuppressants)	Radiotherapy
(1) Localized mass (2) Primary (3) Localized recurrence (4) Young age	(1) Localized mass (2) Renal involvement (3) Recurrent disease	(1) Failure of medical therapy (2) Recurrent mass not responding medically or surgically (3) Unresectable mass

## Abbreviations

ALHE: Angiolymphoid hyperplasia with eosinophilia
CT: Computed tomography
Gy: The gray, SI derived unit
IgE: Immunoglobulin E
IgG: Immunoglobulin G
IgM: Immunoglobulin M
KD: Kimura's disease
MRI: Magnetic resonance imaging
USG: Ultrasound imaging.
